# Molecular characterization of hepatitis B virus X gene in chronic hepatitis B patients

**DOI:** 10.1186/1743-422X-9-131

**Published:** 2012-07-08

**Authors:** Luciana Barbini, Luciana Tadey, Silvina Fernandez, Belen Bouzas, Rodolfo Campos

**Affiliations:** 1 Catedra de Virologia, Facultad de Farmacia y Bioquimica, Universidad de Buenos Aires, Junin 956 4to piso. (1113), Buenos Aires, Argentina; 2 Hospital de Infecciosas F J Muñiz, Buenos Aires, Argentina

**Keywords:** Hepatitis B virus, Genotypes, X protein, Basal core promoter

## Abstract

**Background:**

HBV-X protein is associated with the pathogenesis of HBV related diseases, specially in hepatocellular carcinomas of chronic patients. Genetic variability of the X gene includes genotypic specific variations and mutations emerging during chronic infection. Its coding sequence overlaps important regions for virus replication, including the basal core promoter. Differences in the X gene may have implications in biological functions of the protein and thus, affect the evolution of the disease. There are controversial results about the consequences of mutations in this region and their relationship with pathogenesis. The purpose of this work was to describe the diversity of HBV-X gene in chronic hepatitis patients infected with different genotypes, according to liver disease.

**Methods:**

HBV-X gene was sequenced from chronic hepatitis B patient samples, analyzed by phylogeny and genotyped. Nucleotide and aminoacid diversity was determined calculating intragenetic distances. Mutations at 127, 130 and 131 aminoacids were considered in relation to liver disease.

**Results:**

The most prevalent genotype detected in this cohort was F (F1 and F4), followed by D and A. Most of the samples corresponding to genotypes A and F1 were HBeAg(+) and for genotypes D and F4, HBeAg(−) samples were represented in a higher percentage. Intragenetic distance values were higher in HBeAg(−) than in positive samples for all genotypes, and lower in overlapped regions, compared to single codification ones. Nucleotide and aminoacid diversities were higher in HBeAg(−), than in HBeAg(+) samples.

**Conclusions:**

Independently of the infecting genotypes, mutations at any of 127, 130 and/or 131 aminoacid positions and HBeAg(−) *status* were associated with mild liver disease in this cohort.

## Background

Hepatitis B virus (HBV) belongs to the *Hepadnaviridae* family and can be classified into eight genotypes (A to H). The viral genome has four overlapped open reading frames (ORFs) that codify for: envelope (S/Pre-S), core (C/pre-C), polymerase (P) and X (HBV-X) proteins [1,2]. HBV-X is a 154 aminoacid multifunctional protein with transcriptional transactivator activity on a number of cellular and viral promoters. HBV-X has been associated with the pathogenesis of HBV related diseases, especially in the occurrence of hepatocellular carcinoma in chronic patients [3-6]. The compact nature of the genome and the ORFs overlapping lead to a “constrained evolution” of the virus [7,8]. Genetic variability of the X gene includes genotypic specific variations and mutations emerging during chronic infection [9-14]. Its coding sequence overlaps important regions for virus replication, including the basal core promoter (BCP). Thus, a mutation on the X gene may not only induce aminoacid changes in HBV-X, but also can affect other genes and modify HBV expression [15-17]. In addition, differences in the X gene may have implications in the biological functions of the protein and thus, affect the evolution of the disease [18-21].

During chronic HBV infection, two phases can be distinguished: a first phase characterized by high viral load, HBeAg(+) and high aminotransferases activity; and a second phase with detectable HBV-DNA by PCR, HBeAg(−), detectable anti-HBe antibodies, and normal aminotransferases levels [22,23]. However, there are anti-HBeAg patients who have high ALT levels and detectable serum HBV-DNA, because of BCP mutations.

Natural mutations in the HBV-X gene have been related to the progression of chronic liver disease due to the recession of anti-proliferative and apoptotic effects on the infected hepatocytes, contributing to carcinogenesis [24,25]. Although, there are controversial results about the consequences of mutations in this region and their relationship with pathogenesis [26].

Different studies have failed to find a correlation between BCP mutations (1762–64 nucleotidic positions), viral replication and liver damage in chronic hepatitis B (CHB). The contradictory data may reflect differences between the studied populations (genotype distribution, HBeAg *status*, clinical stage of the patients). The pathogenic role of BCP mutations in CHB and their role in the progression of liver disease is still under debate. Among “hot spot” mutations with impact on HBV-X biological activities, I127T should also be considered [19].

**The aims of this work** were to study the diversity of HBV-X gene and its protein in CHB patients infected with different genotypes, determine the prevalence of 127, 130 and 131 aminoacid mutations and describe their relationship with liver biopsy in an infected cohort of our geographic area.

## Methods

### Patients and samples

Thirty-two serum samples from chronic HBV patients, who were assisted at “Unidad de Hepatopatías Infecciosas” from Hospital Francisco Muñiz, Buenos Aires, were included in this study. Biochemical tests were carried out with routine automated methods. HBsAg, HBeAg, anti-HBe and anti-HBs were measured using commercial kits (MEIA, Axsym, Abbott Laboratories). Serum HBV DNA levels were quantified by an Amplicor HBV Monitor Test (Roche), following the manufacturer´s instructions. Liver biopsy specimens were evaluated according to the modified Knodell score, and categorized as mild or severe fibrosis (F ≤ 2 or F > 2, respectively). All patients were negative for HCV and HIV antibodies. None of the included patients had received antiviral treatment for their chronic hepatitis. This research has complied with all relevant federal guidelines and institutional policies. It was carried out in complaince with the Helsinki Declaration and was approved by the Ethics Committee of the School of Pharmacy and Biochemistry, University of Buenos Aires (Protocol Number: 701283). Written informed consent to participate in this study was obtained from all the patients.

### PCR amplification and sequencing of the X gene

DNA was extracted from 200 μL serum samples with a QIAmp DNA Mini kit (Qiagen Inc., Hilden, Germany), according to the manufacturer´s recommendations. For sequencing , the X region of the genome was amplified by nested PCR. Primers used for the first round were: sense 5´TGCCAAGTGTTTGCTGACGC3´ and antisense 5´ACGGGAAGAAATCAGAAGG3´, and for the second round: sense 5´GCCGATCCATACTGCGGAACT3´ and antisense 5´GGCACAGCTTGGAGGCTTGAA3´. First round PCR was performed with 10 μL of DNA in a 50 μL reaction mixture containing 10X buffer, 200 μM dNTPs, 20 mM Cl_2_Mg, 25 pmol of each primer and 1 U Taq polymerase (Invitrogen). PCR was performed as follows: 94°C 5 min; 94°C 1 min, 45°C 1 min, 72°C 2 min for 45 cycles; and finally 72°C for 10 min. For the second round, 2 μL of the first round PCR product was re-amplified using the same reaction mixture composition, except that internal primers were used. PCR was performed as follows: 94°C 5 min; 94°C 1 min, 55°C 1 min, 72°C 2 min for 40 cycles; and finally 72°C for 10 min. PCR products were run on 1% agarose gel, stained with ethidium bromide, and evaluated under UV light. The amplified products were gel purified using the QIAquick gel extraction kit (Qiagen Inc., Hilden, Germany). The purified products were sequenced by Macrogen, Inc. (Seoul, Korea), using sense and antisense internal primers.

### Phylogenetic analysis

Analysis was performed by comparison of the obtained gene sequences, with HBV reference sequences for each genotype, available at GenBank. HBV-X sequences were aligned using ClustalX software 1.81 version [27]. Phylogenetic trees were constructed using PAUP4b10 package [28] and MODELTEST 3–06 version [29]. Phylogenetic analysis was performed using Maximum Likelihood and Neighbour Joining methods. Robustness of the phylogenetic analysis was evaluated by a 10000 bootstrap resampling. Genetic distances were calculated using the Tamura Nei model of nucleotide substitution, with a gamma distribution of rate heterogeneity between sites, included in MEGA3.1 package. The gamma parameter was determined by using the MODELTEST program.

### Nucleotide sequence accession number

Nucleotide sequences analyzed in this work have been deposited in GenBank under accession numbers: [GenBank: JF911665 to JF911696].

**Table 1:** Patients characteristics, genotypes, nucleotide and aminoacid diversities

**Patient**	**HBeAg*status***	**Viremia level**	**ALT (ULN)**	**Liver disease (histology)**	**Genotype**	**Nucleotides 1762/1764**	**Aminoacids 127/130/131**
**BA54**	+	High	X2	SEVERE	F4	A/G	T/K/V
**BA61**	+	High	X5	MILD	F4	A/G	T/K/V
**BA55**	+	High	X1.5	MILD	F4	A/G	I/K/V
**BA67**	+	High	X3	MILD	F1b	A/G	I/K/V
**BA71**	+	High	X1.5	MILD	F1b	A/G	I/K/V
**BA70**	+	Nd	DTNA	DTNA	F1b	A/G	I/K/V
**BA75**	+	High	X4	SEVERE	F1b	A/G	I/K/V
**BA66**	+	High	X2	SEVERE	F1b	A/G	I/K/V
**BA60**	-	High	X1.2	MILD	F4	A/G	I/K/V
**BA69**	-	Low	X1.7	MILD	F4	T/A	T/M/I
**BA73**	-	Low	X2	MILD	F4	A/G	S/K/V
**BA68**	-	Low	DTNA	DTNA	F4	T/A	S/M/I
**BA58**	-	Nd	DTNA	DTNA	F4	A/G	I/K/V
**BA59**	+	High	X4.5	SEVERE	A	A/G	I/K/V
**BA62**	+	High	X6	MILD	A	A/G	I/K/V
**BA57**	+	High	DTNA	DTNA	A	T/A	I/M/I
**BA56**	+	High	X2	MILD	A	A/G	I/K/V
**BA80**	+	High	X4	SEVERE	A	A/G	I/K/V
**BA72**	-	Low	X5	SEVERE	A	A/G	I/K/V
**BA82**	-	Nd	X2	MILD	A	A/G	I/K/V
**BA78**	-	Low	X1.5	MILD	A	A/A	I/K/I
**BA63**	+	High	X3	MILD	D	C/G	I/T/V
**BA65**	+	High	X1.5	SEVERE	D	A/G	I/K/V
**BA64**	+	High	X3.9	MILD	D	T/A	I/M/I
**BA47**	+	High	X3	MILD	D	A/G	I/K/V
**BA46**	-	High	X2	MILD	D	T/A	I/M/T
**BA50**	-	High	X2.82	SEVERE	D	A/G	T/K/V
**BA49**	-	High	X6.76	SEVERE	D	T/A	T/M/I
**BA76**	-	Low	X2	MILD	D	A/G	L/K/V
**BA77**	-	High	X3	MILD	D	A/A	I/K/I
**BA79**	-	High	X1.5	MILD	D	A/G	L/K/V
**BA81**	-	Low	X4	SEVERE	D	T/G	L/M/V

Main characteristics and results of the 32 patients included in this study. ULN: upper limit of normal levels (41 UI/L); low or high viral loads (Amplicor Monitor Roche): <10^5^ copies/mL / ≥10^5^ copies/mL; nd: not determined; DTNA: data not available; mild or severe histology in liver biopsy specimens were defined according to fibrosis F ≤2 or F >2, respectively, using the modified Knodell score.

**Figure 1:**
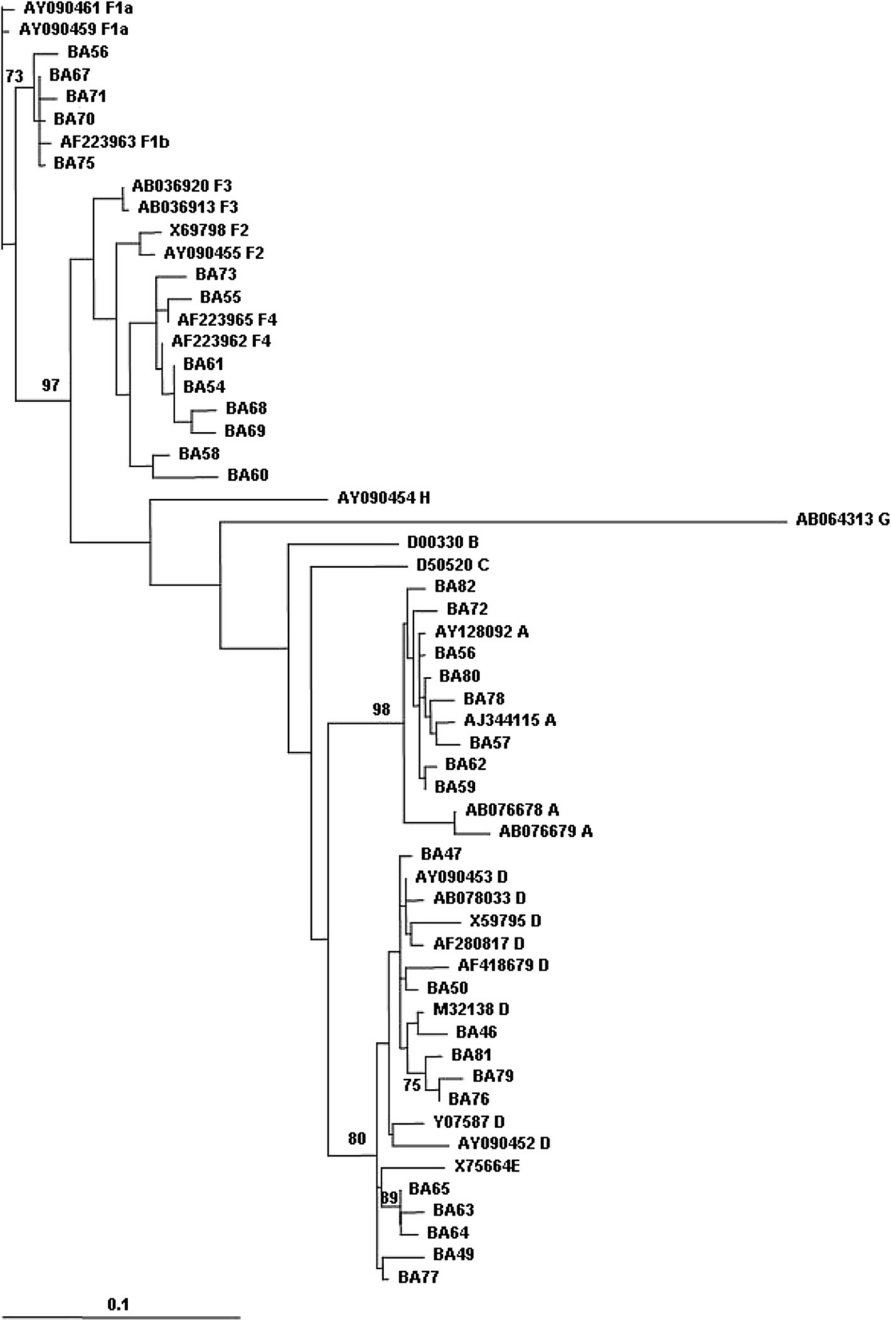
**Phylogenetic tree generated by the samples (BA) included in this study.** GenBank sequences from different genotypes were included as references for the analysis.

## Results and discussion

1. The main characteristics of the studied patients are shown in Table 1.

2. **Phylogenetic analysis and genotyping**To asses genotype distribution in this cohort, genotyping was performed by phylogeny. The phylogenetic analysis grouped the 32 HBV-X gene sequences as: genotype A, 25.0% (n = 8), D 34.4% (n = 11) and F 40.6% (n = 13) (Figure 1). All genotype A sequences were ascribed to subgenotype A2. Genotype F sequences were defined as subgenotype F1b (38.5%, n = 5) and F4 (61.5%, n = 8). Genotype D sequences presented two monophyletic groups corresponding to subgenotype D1 and D3.In summary, the phylogenetic analysis showed that the most prevalent genotype of this sample was F, followed by D and A. These results are in agreement with other previous studies of HBV genotypes distribution in Argentina, in which genotype F, the new world genotype, was the most prevalent, followed by the European genotypes D and A, introduced by migration [30-32].

3. **HBeAg*status*according to genotypes**We assessed the prevalence of HBeAg positive or negative phases during chronic HBV infection, in each genotype. The distribution of HBeAg positive (17) and negative (15) samples, according to their genotype showed that most of the A2 and F1b subgenotypes samples were HBeAg(+): (62.5% (5/8) and 100% (5/5), respectively. On the contrary, HBeAg(−) samples were more abundant for genotypes D and F4 (63.6% (7/11) and 62.5% (5/8), respectively (Table 1). All the analyzed sequences appeared intermingled in the phylogenetic tree, independently from their HBeAg *status*. In summary, genotypes A and F1 showed higher percentage of HBeAg positive samples than negative, and genotypes D and F4 showed higher HBeAg negative than positive samples. The presence of 1858T (characteristic for genotype D) but not 1858C (characteristic for genotype A) facilitates the fixation of the 1896A mutation, stabilizing the encapsidation signal [33-35]. The 1896A mutation abrogates HBeAg expression, establishing the HBeAg(−) phenotype. Interestingly, in this sample F1b subtype resembles to genotype A and F4 subgenotype behaves as genotype D.

4. **Nucleotide sequence analysis and X gene diversity**Distance models tree topology suggests a different diversification for HBeAg(+) and HBeAg(−) sequences. To assess X gene diversity, an analysis of diversification was carried out calculating the intragroup genetic distances (igds). The non-overlapped X gene region with pol gene (nt 1622–1835) and the over-lapped region (nt 1374–1621) within each genotype were analyzed considering their HBeAg *status*. Independently of genotypes, HBeAg(−) samples showed higher igd values than the respective HBeAg(+) ones (Table 2). HBeAg(−) samples present a higher diversity compared to HBeAg(+) samples. This result is in agreement with the idea that mutations are generated during the natural course of infection and HBeAg(−) samples, with longer infection periods, have the opportunity to accumulate more nucleotide changes. In addition, single codification regions present higher igds than double codification regions, suggesting different functional constraints due to the genomic organization of HBV [7,8]. In particular, nucleotides appearing in 1762/1764 positions (BCP) are shown in Table 1. Among all the studied samples (with liver histology data), 8 presented mutations in one or both of these positions. Considering liver disease in patients with mutated X genes, a marked higher proportion of patients with mutations at any of 1762/1764 nucleotides showed mild liver histology (6 out of 8, 75%) and only 2 of 8 (25%) showed severe liver disease. When analyzing the 20 samples with wild type 1762/64 nucleotides, more patients showed mild liver histology (12 out of 20, 60%) than severe liver disease (8 out of 20, 40%), but this difference was not significant. These results can suggest a relation between mutants and slow progression to hepatic disease in this cohort [36].

5. **Amino acid diversity of the X protein, mutations in 127/130/131 positions and HBeAg*status*related to liver disease**To assess X protein diversity, an analysis of diversification was carried out calculating aminoacid divergence values. Total amino acid divergence was determined considering the complete X protein (Table 2). HBeAg(−) samples showed higher aminoacid divergence values, compared to HBeAg(+), in agreement with the obtained nucleotide diversity values, independently of genotypes.

The diversification at the amino acid level was analyzed in particular for 127, 130 and 131 positions. Aminoacid diversities at these positions in all groups are shown in Table 1. The samples that present mutations at any of the three studied positions (14/28 with liver histology data) were more associated with mild biopsies (10/14, 71%) than to severe ones (4/14, 29%). When analyzing the 14 samples with wild type amino acids, more patients showed mild liver histology (8 out of 14, 57%) than severe liver disease (6 out of 14, 43%) but the differences between groups were not so pronounced as in the mutant samples.

**Table 2:** Nucleotide and aminoacid divergences

**n**	**Group**	**X gene(Non-overlapped region)**	**X gene(Over-lapped region with pol gene)**	**CompleteX protein**
		**% nucleotide divergence**	**% nucleotide divergence**	**% aminoacid divergence**
		**Mean ± SD**	**Mean ± SD**	**Mean ± SD**
3	F4 HBeAg(+)	1.9 ± 0.9	0.6 ± 0.4	2.28 ± 1.01
5	F4 HBeAg(−)	5.7 ± 1.7 ^(*)^	3.2 ± 0.9 ^(*)^	7.00 ± 1.54 ^(*)^
5	F1b HBeAg(+)	1.4 ± 0.6	0.7 ± 0.3	1.89 ± 0.70
4	D HBeAg(+)	1.9 ± 0.7	2.5 ± 0.7	3.54 ± 0.96
7	D HBeAg(−)	4.4 ± 1.2 ^(*)^	2.1 ± 0.6	5.66 ± 1.35 ^(*)^
5	A HBeAg(+)	1.3 ± 0.6	0.8 ± 0.4	2.04 ± 0.77
3	A HBeAg(−)	1.9 ± 0.9	2.1 ± 0.8 ^(*)^	3.43 ± 1.32 ^(*)^

Genetic distances within genotypes (HBeAg *status* + or -) in overlapped and non-overlapped regions (analyzing nucleotides), and in the whole X protein (aminoacids). ^(*)^ denotes statistically significant differences between HBeAg +/− groups within each genotype.

Independently of the HBeAg *status*, higher percentages of mild liver pathology than severe one was observed in both groups: 9/15 (60%) for HBeAg(+) and 9/13 (69%) for HBeAg(−). When considering together the HBeAg *status*, mutants and liver pathology, the wild type HBeAg(+) group showed similar numbers for mild or severe hepatic histology (6 and 5 out of 11, respectively). But, for the mutant HBeAg(−) group, 7 out of 10 (70%) presented mild biopsies (Table 3).

**Table 3:** **Wild type/mutants and HBeAg*status*related to liver disease**

	**Severe**	**mild**	**DTNA**
**Wild type**	6	8	2
**Mutants**	4	10	2
	**Wild type**	**severe**	**mild**
**HBeAg(+)**	11	5	6
**HBeAg(−)**	3	1	2
	**Mutants**	**severe**	**mild**
**HBeAg(+)**	4	1	3
**HBeAg(−)**	10	3	7

X protein aminoacid diversity at 127/130/131 positions (wild type/mutants) and HBeAg *status* (+/−) related to liver disease (mild/severe, according to the modified Knodell score, F ≤2 or F >2, respectively). DTNA: data not available.

These results suggest that in this particular cohort, mutations at the studied positions, associated with a HBeAg(−) *status*, give certain protection to evolve to more severe liver pathology, suggesting protective roles in the progression of disease [37]. The results obtained in this work and in this particular studied population do not agree with previous findings that associated 127, 130 and 131 X protein mutations with severity of disease [38-42]. These findings justify further similar studies in large cohorts of patients.

## Conclusions

In conclusion, the distribution of HBV genotypes in the studied samples showed genotype F as the most prevalent, followed by D and A. Most of A2 and F1 subgenotype samples were HBeAg(+), but for D and F4 genotypes HBeAg(−) samples were more abundant. Considering X gene and protein variability, HBeAg(−) samples showed higher diversity compared to positive samples. Finally, samples with mutations at any of 127, 130 and/or 131 aminoacid positions and HBeAg(−) *status* could be associated with mild liver disease*.*

## Abbreviations

ALT, Alanine transaminase; BCP, Basal core promoter; CHB, Chronic hepatitis B; C, Core; DNA, Deoxyribonucleic acid; dNTPs, Deoxyribonucleotide triphosphates; HBeAg, Hepatitis B virus “e” antigen; HBsAg, Hepatitis B virus “s” antigen; HBV, Hepatitis B virus; HBV-X, Hepatitis B virus X protein; HCV, Hepatitis C virus; HIV, Human immunodeficiency virus; Igds, Intragenetic distances; nt, Nucleotide; P, Polymerase; S, Surface; PCR, Polymerase chain reaction.

## Competing interest

The authors declare they have no competing interests.

## Authors’ contributions

LB contributed to the conception and the design of the study, acquisition of data, analysis of results, writing of the manuscript and its final approval; LT contributed to the analysis of the results; SF and BB contributed to the design of the study, RC contributed to the conception and the design of the study, analysis of the results, writing the manuscript and its final approval. All authors read and approved the final manuscript.
